# Microbial Mechanistic Insight into the Role of Inulin in Improving Maternal Health in a Pregnant Sow Model

**DOI:** 10.3389/fmicb.2017.02242

**Published:** 2017-11-17

**Authors:** Pan Zhou, Yang Zhao, Pan Zhang, Yan Li, Taotao Gui, Jun Wang, Chao Jin, Lianqiang Che, Jian Li, Yan Lin, Shengyu Xu, Bin Feng, Zhengfeng Fang, De Wu

**Affiliations:** Key Laboratory of Animal Disease–Resistance Nutrition and Feed Science, Ministry of Agriculture, People's Republic of China, Animal Nutrition Institute, Sichuan Agricultural University, Chengdu, China

**Keywords:** dietary fiber, gestation, microbial composition, maternal health, neonatal body mass index

## Abstract

General consumption of “western diet” characterized by high refined carbohydrates, fat and energy intake has resulted in a global obesity epidemics and related metabolic disturbance even for pregnant women. Pregnancy process is accompanied by substantial hormonal, metabolic and immunological changes during which gut microbiota is also remarkably remodeled. Dietary fiber has been demonstrated to have a striking role in shifting the microbial composition so as to improve host metabolism and health in non-pregnant individuals. The present study was conducted to investigate effects of adding a soluble dietary fiber inulin (0 or 1.5%) to low- or high- fat (0 or 5% fat addition) gestational diet on maternal and neonatal health and fecal microbial composition in a sow model. Results showed that inulin addition decreased the gestational body weight gain and fat accumulation induced by fat addition. Circulating concentrations of pro-inflammatory cytokine IL-6, adipokine leptin and chemerin were decreased by inulin supplementation. Inulin addition remarkably reduced the average BMI of newborn piglets and the within litter BMI distributions (%) ranging between 17 and 20 kg/m^2^, and increased the BMI distribution ranging between 14 and 17 kg/m^2^. 16S rRNA gene sequencing of the V3-V4 region showed that fecal microbial changes at different taxonomic levels triggered by inulin addition predisposed the pregnant sow to be thinner and lower inflammatory. Meanwhile, fecal microbial composition was also profoundly altered by gestation stage with distinct changes occurring at perinatal period. Most representative volatile fatty acid (VFA) producing-related genera changed dramatically when reaching the perinatal period and varied degrees of increases were detected with inulin addition. Fecal VFA concentrations failed to show any significant effect with dietary intervention, however, were markedly increased at perinatal period. Our findings indicate that positive microbial changes resulted by 1.5% soluble fiber inulin addition would possibly be the potential mechanisms under which maternal body weight, metabolic and inflammatory status and neonatal BMI were improved. Besides, distinct changes of microbial community at perinatal period indicated the mother sow is undergoing a catabolic state with increased energy loss and inflammation response at that period compared with other stages of gestation.

## Introduction

Rapid development of modern food industry drives people to change their dietary habits across the world. Trends in consumption of energy-dense diet containing highly-refined carbohydrates, fat and low dietary fiber are accompanied by a global obesity epidemics and related chronic metabolic diseases (Popkin et al., 2012). As for gestating women, diet-induced obesity or excessive gestational weight gain could not only result in remarkable influences on the maternal health and pregnancy outcomes, but also long-term effects on their offspring (Fraser et al., 2010; Lawlor et al., 2012). Gestation period is a key window for both mother and offspring with tremendous hormonal, metabolic and immunological changes occurring in the maternal body (Newbern and Freemark, 2011), during which microbiota remodeling is thought to be a positive response for mother to support a successful pregnancy (Mandal et al., 2016). Gut microbiota, living with an intimate relationship with its host, has been identified as major regulator in host nutrients metabolism, gastrointestinal health and immunologic functions (Hooper et al., 2012).

Previous studies have highlighted dietary fiber supplementation as an effective manipulation to improve diet-induced obesity and related metabolic abnormalities (Brownlee, 2011; Chen et al., 2015). As the major energy source for gut microbiota, dietary fiber is believed to have significant effects on the composition and diversity of microbiota (De Filippo et al., 2010; Brownlee, 2011; Heinritz et al., 2016). The awareness about the effect of microbiota on host metabolism and health has provided insights about the role of gut microbiota and their metabolites, short chain fatty acids (SCFA), in the link between dietary fiber and obesity and its related metabolic syndromes (Delzenne and Cani, 2011; den Besten et al., 2013a). However, due to the various physicochemical properties of dietary fiber, physiological effects of dietary fiber also vary greatly as reviewed by Hamaker and Tuncil (2014). Soluble dietary fiber, in particular, which is easily fermented, would have greater impact on bacterial metabolism compared with insoluble dietary fiber (Gråsten et al., 2002). Inulin-type fructans, a typical soluble dietary fiber, is a mixture of polymers and oligomers, which are composed of fructosyl units linked by β(2 → 1) glycosidic bonds. Due to this β-configuration, inulin is resistant to hydrolysis by digestive enzymes (Micka et al., 2017).

Given that there are few studies aiming to evaluate the microbial mechanism of soluble dietary fiber in improving maternal and neonatal health upon different diet types during pregnancy, the current research was undertaken to investigate effects of adding inulin to low- or high -fat diet, on the composition and metabolites of fecal microbiota from early gestation to perinatal period, as well as maternal and neonatal health parameters in a pregnant sow model. It is supposed to provide some microbial mechanistic insights into the application of inulin to a typical gestational diet characterized by high fat and energy intake for improving maternal and neonatal health.

## Materials and methods

### Ethical approval

The research protocol was approved by the Care and Use committee of Sichuan Agricultural University under ethic approval number DKY-B20121602.

### Animals and experimental design

A total of 20 Landrace × Yorkshire fifth parity sows with similar body weight (BW) and backfat were used. Sows were inseminated with semen from the same Duroc boar. After insemination, sows were then allocated to one of four treatments as a 2 × 2 experimental design according to their backfat thickness and BW. The four treatments were low fat diet (LFD; without soybean oil added), LFD with 1.5% inulin (LFD.Inu), high fat diet (HFD; 5% soybean oil added) and HFD with 1.5% inulin (HFD. Inu).

During gestation, all sows were fed the same amount of feed during the whole gestation. In detail, sows were fed 2.3 kg/d of corresponding diet from d0 to 90 of gestation and 2.80 kg/d diet from d91 to parturition. Sows were fed twice per day at 0800 and 1600 h. On d107 of pregnancy, sows were moved to individual farrowing pen. Sows had free access to water during the experiment. The average ambient temperature in the gestation house was maintained at 22–26°C.

### Diets and ingredients

Ingredient and nutrient composition of experimental diets were presented in Table S1. All diets based on corn-soybean meal were formulated to meet or exceed the nutrients requirements of gestating sows as recommended by the National Research Council (2012) and to contain same content for all nutrients other than carbohydrates and lipids. The inulin used in the study was obtained from BENEO-Orafti (Orafti GR, Belgium) with purity >90% and average degree of polymerization (DP) = 10–12.

### Sow feed intake, body weight and backfat measurements during gestation

Food intake was recorded daily before morning meal. Sow fasting body weight and backfat thickness were measured at mating, d30, 60, 90, and 112 of gestation as well as the day after farrowing. The backfat thickness was measured at 65 mm to the left side of the dorsal mid-line at the last rib (P2) using ultrasound scanner (Renco Lean-Meater; Renco Corporation, Minneapolis, MN, USA).

### Sow backfat biopsy and adipokines analyses

A backfat biopsy was obtained from each sow on d105 of gestation. Sows were anesthetized with an intramuscular injection of combined anesthetics named Shumianning (compounds of ketamine, xylazine and midazolam; does as 1 ml/ 80 kg body weight, Nanjing Agricultural University, Jiangsu, China). A backfat sample was collected at P2 point of the right side. Samples were immediately frozen in liquid nitrogen and stored at −80°C until analysis.

Adipose tissues were homogenized with cold 0.9% saline solution (W/V: 1:9, g/mL) in an ice-water bath. The homogenate was centrifuged at 4°C at 1,500 × g for 10 min. The fat layer was removed, and the remanent supernatant was analyzed for leptin, adiponectin and chemerin. These three hormones were measured with commercial porcine enzyme-linked immunosorbent assay kits according to the manufacturer's instructions (Nanjing Jiancheng Institute of Bioengineering, China).

### Analyses of maternal blood inflammatory indices and adipokines at perinatal period

Fasting blood samples (10 ml) were collected from each sow per treatment before morning meal on d110 of gestation. Blood were collected into two tubes containing no anticoagulant and left at room temperature for 2 h followed by centrifuging for 10 min at 2,550 × g at 4°C. Serum samples were harvested and stored at −20°C until analysis.

Serum pro-inflammatory index IL-6, anti-inflammatory index IL-10, leptin, adiponectin and chemerin were measured with commercial porcine enzyme-linked immunosorbent assay kits according to the manufacturer's instructions (Nanjing Jiancheng Institute of Bioengineering, China).

### Body mass index (BMI) distribution of neonatal piglets

At birth, birth weight and crown–rump length (CRL, the supine length of the piglet from the crown of its head to the base of its tail) of neonatal piglets were measured. Body mass index [BMI; birth weight/(crown–rump length)^2^] were calculated for each piglet as described by Baxter et al. (2008).

### Fecal metabolites and microbial analyses

Fresh feces of sows who did not have disease and diarrhea before sampling were collected in duplicate into two sterile tubes and kept on ice until transferring them to a freezer at −80°C within 10 min in the morning immediately after defecation at d30, 60, 90, and 110 of gestation, respectively.

One of the duplicate samples was analyzed for pH and VFA (acetate, propionate and butyrate) concentration. The pH values were measured according to Topping et al. (1993) with some modifications. Briefly, 0.5 g of feces was diluted with distilled water as the ratio of 1:2 (weight/volume) and homogenized for 60 s in a blender. Then the homogenate was centrifuged (3,000 × g, 15 min, 20°C), and measured with a pH meter (PHS-3C pH, Shanghai, China). The VFA concentrations were measured using a gas chromatographic method as described by Chen et al. (2013) with minor modifications. Briefly, 2 g of fecal sample was suspended in 5 ml of distilled water and placed for 30 min. Afterwards, the sample was centrifuged (12,000 × g) at 4°C for 10 min. The 2 ml supernatant was transferred and mixed with 0.4 ml metaphosphoric acid. After 30 min at 4°C, the sample was centrifuged (12,000 × g) again at 4°C for 10 min. The supernatant (1.2 ml) was transferred and mixed with 15.2 μl crotonic acid (210 mmol/L, internal standard), then 0.3 ml liquid was transferred and mixed with 0.3 ml methanol. Aliquot of the supernatant (1 μl) was analyzed using a gas chromatography (Varian CP-3800 GC, USA).

Another sample was used for microbial analysis. Microbial DNA was extracted from 0.25 g of thawed stool samples using the Mo Bio PowerFecal^TM^ DNA Isolation Kit (MO BIO Laboratories, Carlsbad, CA, USA) according to the manufacturer's protocol. Before sequencing, the concentration and purity of the extracted genomic DNA were measured. The integrity of the extracted genomic DNA was determined by electrophoresis on a 1% (w/v) agarose gel. Extracted fecal DNA samples were sent to Novogene Bioinformatics Technology (Beijing, China) to perform amplicon pyrosequencing on the Illumina HiSeq PE250 platforms. The V4 hypervariable region of the 16S rRNA gene was amplified using 515F and 806R primer (5′-GTGCCAGCMGCCGCGGTAA-3′ and 5′-GGACTACHVGGGTWTCTAAT-3′, respectively).

The effective tags were mapped to OTUs using Uparse v7.0.1001 at 97% sequence similarity. Representative sequences for each OTU were selected. The Ribosomal Database Project (RDP) classifier Version 2.2 was used to assign a taxonomic rank to each representative sequence. The relative abundance of each OTU was examined at different taxonomic levels. Diversity within communities (Alpha diversity) calculations and taxonomic community assessments were performed by Qiime 1.7.0.

### Statistical analysis

Sows and their litters were regarded as the experimental units. One sow from LFD group had an unexplained diarrhea on d88 of gestation which lasted for 2 days, therefore, its fecal samples for VFA and microbial analyses on d90 and d110 of gestation were excluded from the present study. Data of relative abundance at phylum and genus level were log-transformed before statistical analysis, while data of relative abundance of representative VFA-producing related genera were log-transformed following the addition of a small offset (0.00001) to counteract the presence of zero values before statistical analysis. The concentrations of total and individual VFA, Alpha diversity index (Chao 1 index and Simpson index) and log-transformed relative abundances at different taxonomic levels were applied to the following model using MIXED procedure of SAS (version 9.3; SAS Inst Inc., Cary, NC) to analyze data:

$$\begin{array}{l} Y_{ijkl}=\mu +\alpha_{i}+\beta_{j}+\gamma_{k}+{\left(\alpha \beta \gamma \right)}_{ijk}+t_{l}+\varepsilon_{ijkl} \end{array}$$

Where Y_*ijkl*_ is the response variable, μ is the overall mean, α_*i*_, β_*j*_, and γ_*k*_ are the fixed effects of dietary fat level (*i* = LFD, HFD), dietary inulin level (*j* = 0% inulin, 1.5% inulin) and gestation stage (*k* = G30, G60, G90, G110), respectively. (αβγ)_*ijk*_ is the interaction among fixed effects, *t*_*l*_ is the random effect of sows to account for repeated measurements within sow and ε_*ijkl*_ is the residual error. Other variables except for those mentioned above were analyzed with a similar model without effects of gestation stage and repeated measurements.

Values were expressed as mean + largest SEM in tables and as means ± SEM in figures, except that confidence limits were given in brackets instead of SEM values for data of relative abundances at different taxonomic levels. *P* ≤ 0.05 was considered statistically significant, whereas 0.05 < *P* < 0.1 were considered as showing a trend. When significant main effects or interative effects were observed, the means were compared using the least significant difference method with a *P* < 0.05 indicating significance.

## Results

### Feed intake and phenotype changes during gestation

**Figure 1:**
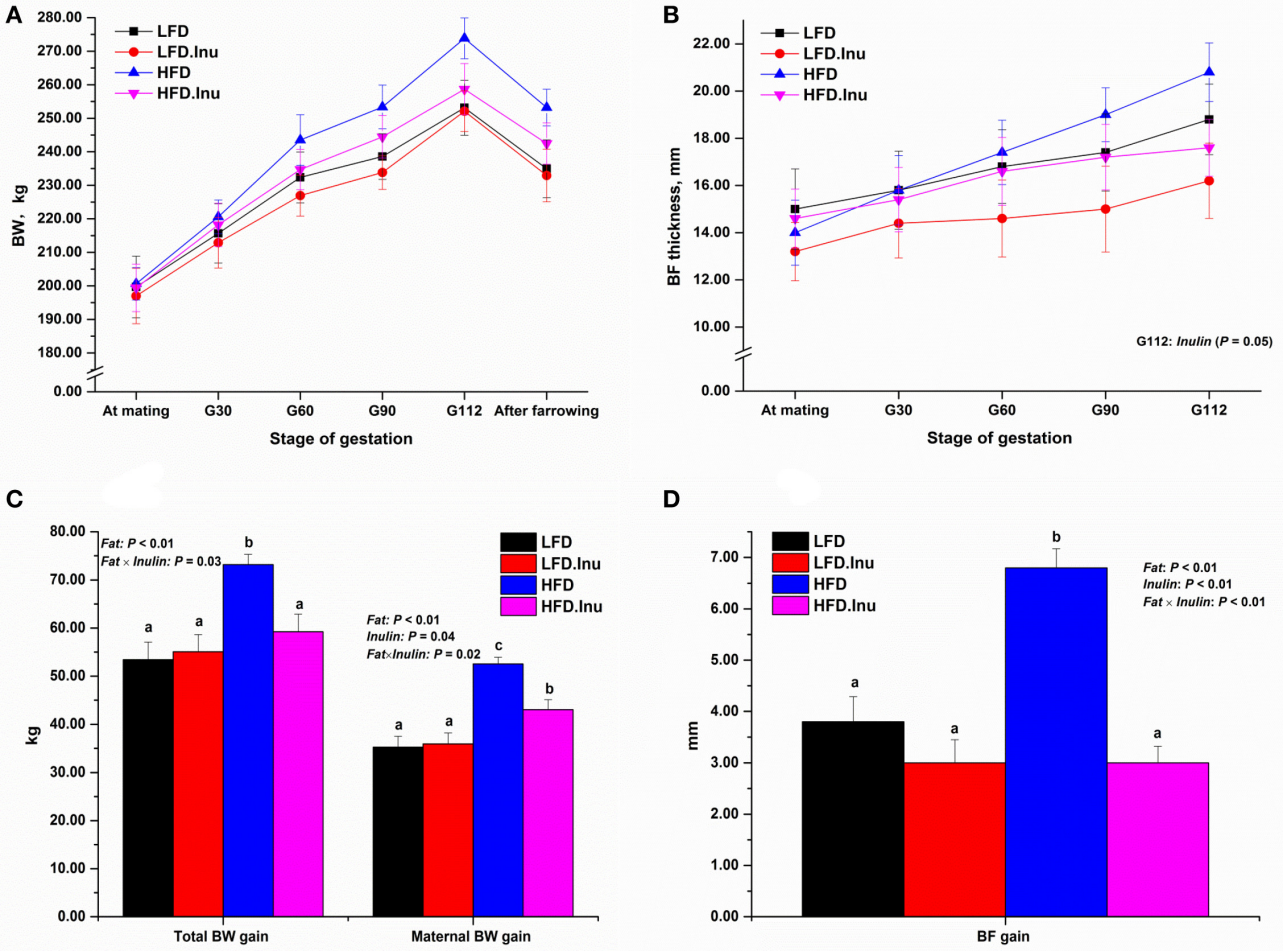
**(A,C)** Body weight (BW) and **(B,D)** Backfat (BF) changes during gestation. Data were expressed as means ± SEM. Sows were regarded as the experimental units, *n* = 5 for each treatment. **(B)** BF thickness on G112: *P* = 0.05 for inulin effect. **(C)** Total BW gain: *P* < 0.01, = 0.08, and = 0.03 for fat, inulin and fat × inulin interaction effect, respectively; Maternal BW gain: *P* < 0.01, = 0.04, and = 0.02 for fat, inulin and fat × inulin interaction effect, respectively. **(D)** BF gain: *P* < 0.01 for fat, inulin, and fat × inulin interaction effect, respectively. When significant main effects or interative effects were observed, the means were compared using the least significant difference method with a *P* < 0.05 indicating significance. Therefore, mean values without a common letter are significantly different for each parameter in the figure (*P* < 0.05). LFD, low fat diet; LFD.Inu, low fat diet with inulin addition; HFD, high fat diet; HFD.Inu, high fat diet with inulin addition; maternal BW gain, net weight gain of sow itself. Only significant *p*-values were presented in the figure.

During the whole gestation, sows from all groups consumed their daily feed completely and no feed residue was recorded. As a result, sows with high fat treatment consumed more energy, fat content than their counterparts. Sow body weight and backfat changes during gestation were shown in Figure 1. From mating to parturition, body weight and backfat thickness did not differ (*P* > 0.05) between treatments at any time point, except that inulin addition significantly reduced the backfat thickness on d112 of gestation compared to non-inulin addition group (16.20 vs. 18.80, *P* = 0.05). In view of the changes for the whole gestation, fat addition dramatically increased the total BW gain (+22%), maternal BW gain (+34%), and backfat gain (+44%; *P* < 0.01). On the contrary, inulin addition reduced maternal BW gain (−10%; *P* = 0.04) and backfat gain (−43%; *P* < 0.01), and also showed remarkable interactive effects with fat level on total BW (*P* = 0.03), maternal BW (*P* = 0.02) and backfat gain (*P* = 0.02). These interactive effects indicated the lowering effects of inulin were prominent in HFD.Inu group with 19, 18, and 56% reduction for total BW, maternal BW and backfat gain, respectively compared to HFD group (*P* < 0.05).

### Changes in the concentration of maternal blood inflammatory indices and adipokines in serum and backfat tissues at perinatal period

**Figure 2:**
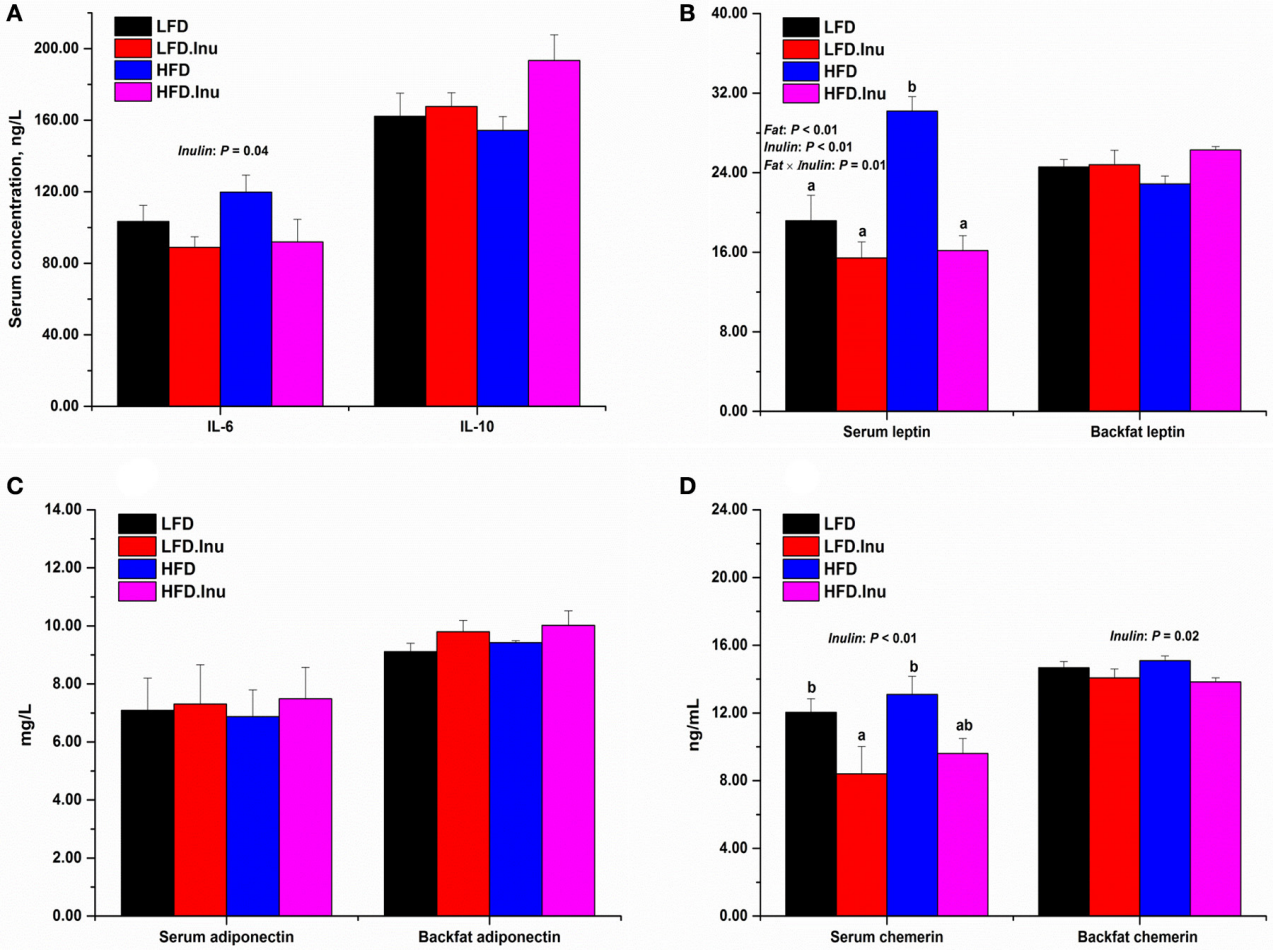
Maternal **(A)** serum inflammatory indices and **(B–D)** adipokines at perinatal period. Data were expressed as means ± SEM. Sows were regarded as the experimental units, *n* = 5 for each treatment. **(A)** IL-6: *P* = 0.04 for inulin effect; IL-10: *P* = 0.06 for inulin effect. **(B)** Serum leptin: *P* < 0.01 for fat and inulin effect and *P* = 0.01 for fat × inulin interaction effect; Backfat leptin: *P* = 0.06 for inulin effect. **(C)** Backfat adiponectin: *P* = 0.08 for inulin effect. **(D)** Serum chemerin: *P* < 0.01 for inulin effect; Backfat chemerin: *P* = 0.02 for inulin effect. When significant main effects or interative effects were observed, the means were compared using the least significant difference method with a *P* < 0.05 indicating significance. Therefore, mean values without a common letter are significantly different for each parameter in the figure (*P* < 0.05). LFD, low fat diet; LFD.Inu, low fat diet with inulin addition; HFD, high fat diet; HFD.Inu, high fat diet with inulin addition. Only significant *p*-values were presented in the figure.

As shown in Figure 2A, inulin addition significantly decreased the concentration of serum pro-inflammatory cytokine IL-6 (90.48 vs. 111.60 ng/L, *P* = 0.04), and tended to increase that of anti-inflammatory cytokine IL-10 (180.53 vs. 158.23 ng/L, *P* = 0.06). As for the adipokines (Figures 2B–D), fat addition increased the serum leptin (23.18 vs. 17.30 ng/mL, *P* < 0.01), while inulin resulted in a remarkable reduction in it (15.80 vs. 24.69 ng/mL, *P* < 0.01). Interactive effect between fat and inulin (*P* = 0.01) was also found for serum leptin indicating an improving effect of inulin addition upon high fat treatment. Inulin addition showed a tendency to increase the concentrations of backfat leptin (25.55 vs. 23.72 ng/mL, *P* = 0.06) and backfat adiponectin (9.91 vs. 9.26 mg/L, *P* = 0.08). Inulin addition significantly decreased the concentrations of serum chemerin (9.00 vs. 12.57 ng/mL, *P* < 0.01) and backfat chemerin (13.95 vs. 14.88 ng/mL, *P* = 0.02).

### Body mass index distribution of neonatal piglets

The BMI distribution of neonatal piglets was shown in Table 1. Inulin addition remarkably decreased the average BMI of newborn piglets (*P* < 0.01). The BMI distributions (%) ranging between 14 and 17 and between 17 and 20 kg/m^2^ with inulin addition were 37.7% higher (91.42 vs. 53.68%, *P* < 0.01) and 38.6% lower (3.63 vs. 42.25%, *P* < 0.01) than those from inulin-free group, respectively.

**Table 1:** Effects of inulin addition to low- or high –fat diets on body mass index (BMI) distribution of neonatal piglets.

	**Treatments**				**SEM**	***P*****-value**		
	**LFD**	**LFD.Inu**	**HFD**	**HFD.Inu**		**Fat level**	**Inulin level**	**Fat × Inulin**
Average BMI, kg/m^2^	16.45^b^	15.77^a^	16.89^b^	15.58^a^	0.20	0.55	<0.01	0.14
BMI distribution,%								
BMI < 14 kg/m^2^	4.78	2.76	3.36	7.14	2.57	0.57	0.74	0.28
14 < BMI < 17 kg/m^2^	60.86^a^	94.27^b^	46.51^a^	88.57^b^	6.85	0.16	<0.01	0.54
17 < BMI < 20 kg/m^2^	34.36^b^	2.97^a^	50.13^b^	4.29^a^	7.08	0.25	<0.01	0.32

*Data are expressed as mean ± largest SEM. Sows and their litters were regarded as the experimental units, n = 5 for each treatment. When significant main effects or interative effects were observed, the means were compared using the least significant difference method with a P < 0.05 indicating significance. Therefore, within a row and within a main effect, mean values without a common letter are significantly different (P < 0.05). LFD, low fat diet; LFD.Inu, low fat diet with inulin addition; HFD, high fat diet; HFD.Inu, high fat diet with inulin addition*.

### Changes of fecal PH and microbial metabolites VFAs

As shown in Table 2, fat and inulin addition failed to show any remarkable effects on VFA concentrations in spite of numerical increases were found. By contrast, fat and inulin addition markedly decreased the pH values (*P* < 0.01 and *P* = 0.04, respectively). Numerical increases of VFA concentrations and significant decreases of pH values caused by fat addition were largely due to HFD.Inu group as shown in Figure S1, and also could be indicated by the significant interactions between fat and inulin in Table 1 (*P* ≤ 0.01, respectively). Gestation stage had noteworthy effects on concentrations of total and individual VFA as well as the pH value. Total and individual VFA concentrations decreased linearly from d30 to d90 of gestation, but, interestingly, rose again on d110 of gestation.

**Table 2:** Effects of inulin addition to low- or high –fat diets on fecal VFA concentrations of gestating sows.

	**Fat level**		**Inulin level**		**Gestation stage**				**SEM**	***P*****-value**					
	**Low**	**High**	**0**	**1.5%**	**d30**	**d60**	**d90**	**d110**		**F**	**I**	**S**	**F × I**	**F × S**	**I × S**
PH value	6.09^b^	5.97^a^	6.08^b^	5.99^a^	5.99^a^	6.01^a^	6.17^b^	5.95^a^	0.04	<0.01	0.04	<0.01	<0.01	0.24	0.03
Acetate, μmol/g	54.91	60.18	56.59	58.51	59.81^bc^	55.36^ab^	47.05^a^	67.96^c^	3.45	0.13	0.57	<0.01	<0.01	0.59	0.30
Propionate, μmol/g	15.16	16.98	15.59	16.55	17.17^bc^	14.74^ab^	12.60^a^	19.77^c^	1.10	0.10	0.38	<0.01	<0.01	0.88	0.37
Butyrate, μmol/g	7.01	7.71	6.95	7.77	7.81^abc^	6.83^ab^	6.06^a^	8.75^c^	0.71	0.32	0.24	0.05	0.01	0.94	0.63
Total VFA, μmol/g	77.08	84.88	79.12	82.84	84.79^bc^	76.94^ab^	65.71^a^	96.48^c^	4.79	0.10	0.43	<0.01	<0.01	0.68	0.28

*Data are expressed as mean ± largest SEM. Sows were regarded as the experimental units, n = 5 for each treatment at each gestation stage except for LFD group with n = 4 on d90 and d110 of gestation due to an unexplained diarrhea on d88 of gestation which lasted for 2 days. When significant main effects or interative effects were observed, the means were compared using the least significant difference method with a P < 0.05 indicating significance. Therefore, within a row and within a main effect, mean values without a common letter are significantly different (P < 0.05). F, fat level; I, inulin level; S, gestation stage, F × I, interaction between fat and inulin level; F × S, interaction between fat and gestation stage; I × S, interaction between inulin and gestation stage; VFA, volatile fatty acid. F × I × S interactions were tested but were not significant (P > 0.10)*.

### Changes of fecal microbial diversity

**Figure 3:**
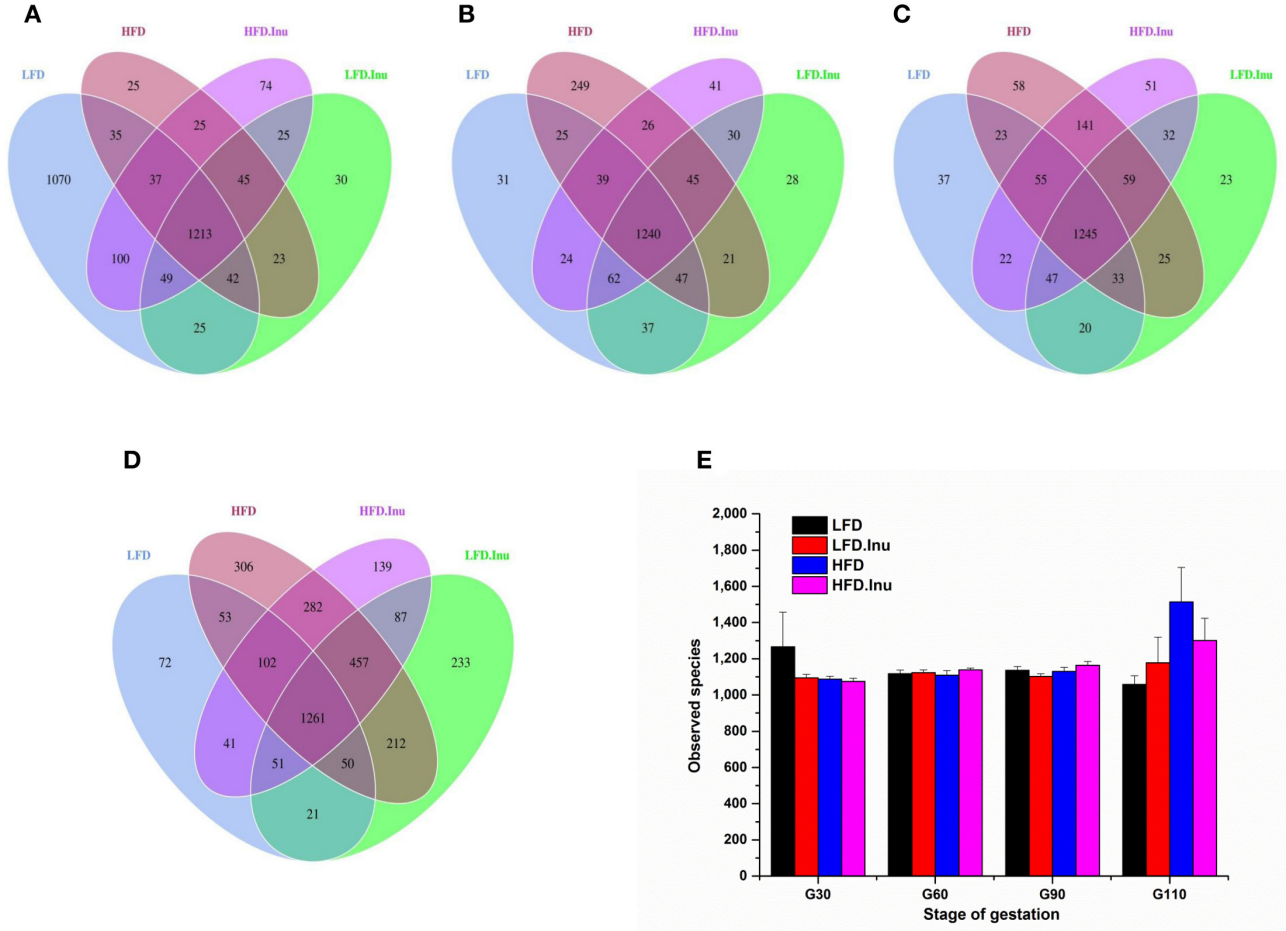
Comparison of the OTUs among treatments at each gestation stage. The observed OTUs sharing ≥97% sequence similarity. (**A–D**) Venn diagrams were generated to describe the common and unique OTUs among treatments at d30, 60, 90, and 110 of gestation, respectively. **(E)** Observed species at different gestation stage. Sows were regarded as the experimental units, *n* = 5 for each treatment at each gestation stage except for LFD group on d90 and d110 with *n* = 4 due to an unexplained diarrhea on d88 of gestation which lasted for 2 days. LFD, low fat diet; LFD.Inu, low fat diet with inulin addition; HFD, high fat diet; HFD.Inu, high fat diet with inulin addition.

Average raw reads, average effective tags and average OTUs for each treatment during gestation were shown in Table S2. At each gestation stage, LFD-HFD and HFD.Inu–LFD.Inu pairs shared more common OTUs with each other, respectively. Common OTUs for LFD-HFD and HFD.Inu–LFD.Inu pairs increased from 1,327 and 1,332 on d30 of gestation to 1,466 and 1,856 on d110 of gestation, respectively (Figures 3A–D). There was no dietary effect or interaction effect on the numbers of observed species at each gestation stage (Figure 3E).

**Figure 4:**
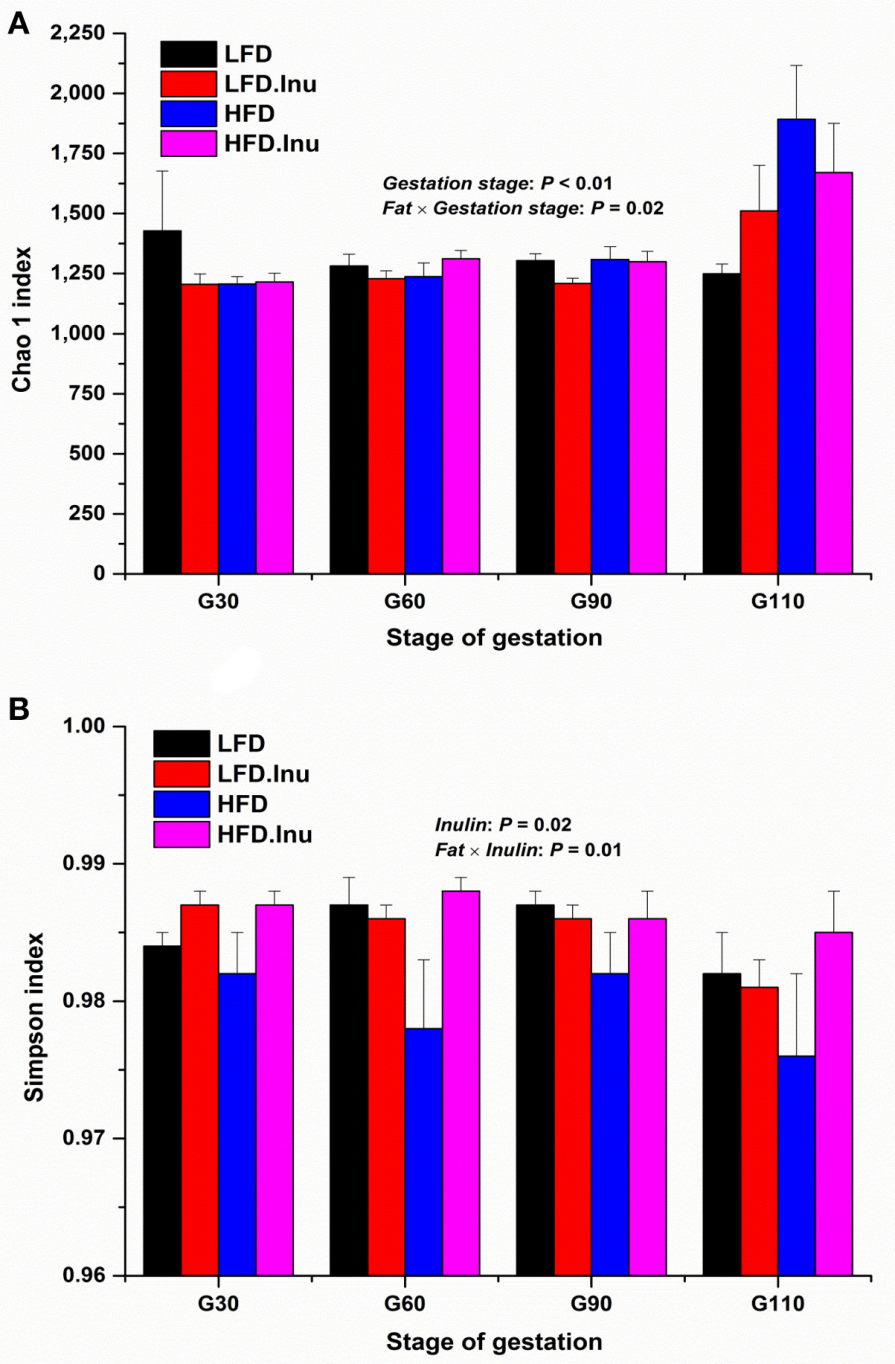
**(A)** Chao 1 and **(B)** Simpson index analyses over the course of gestation. Sows were regarded as the experimental units, *n* = 5 for each treatment at each gestation stage except for LFD group on d90 and d110 with *n* = 4 due to an unexplained diarrhea on d88 of gestation which lasted for 2 days. **(A)** Chao 1 index: *P* < 0.01 and *P* = 0.02 for gestation stage and fat × gestation stage interaction effect, respectively. **(B)** Simpson index: *P* = 0.02 and *P* = 0.01 for inulin and fat × inulin interaction effect, respectively. When significant main effects or interative effects were observed, the means were compared using the least significant difference method with a *P* < 0.05 indicating significance. Therefore, mean values without a common letter are significantly different for each parameter in the figure (*P* < 0.05). LFD, low fat diet; LFD.Inu, low fat diet with inulin addition; HFD, high fat diet; HFD.Inu, high fat diet with inulin addition. Only significant *p* values were presented in the figure.

To assess fecal microbial community structure, richness (Chao 1 index) and diversity (Simpson index) were calculated (Figure 4). For the Chao 1 index, gestation stage exhibited significant effect on it with data on d110 of gestation being much higher than any other stages (*P* < 0.01). There was an interactive effect between fat level and gestation stage (*P* = 0.02). A remarkable increment in Simpson index with inulin supplementation was found in the present study (*P* = 0.02). Interative effect between fat and inulin level on Simpson index (*P* = 0.01) was also detected to provide the view that inulin addition could effectively improve the decline of Simpson index induced by high fat addition.

### Changes of the relative abundance at phylum level

The relative abundances at phylum level of all samples during gestation were present in Figure S2, suggesting that the top five dominated phyla were *Firmicutes, Bacteroidetes, Spirochaetes, Tenericutes*, and *Proteobacteria*. Nine phyla (>1% in at least one sample) and *Firmicutes*/*Bacteroidetes* ratio were chosen for significance analyses (Table 3). High fat treatment decreased the relative abundance of *Proteobacteria* (*P* = 0.04), tended to decrease the relative abundance of *Bacteroidetes* (*P* = 0.06), and tended to increase the relative abundance of *Actinobacteria* (*P* = 0.08) and *Firmicutes/Bacteroidetes* ratio (*P* = 0.07). Inulin supplementation increased the relative abundance of *Lentisphaerae* (*P* = 0.02), and tended to decrease the relative abundance of *Tenericutes* (*P* = 0.09). All phyla were significantly affected by gestation stage (*P* < 0.05, respectively) indicating that they varied a lot from early, even from late gestation, to perinatal period. In particular, relative abundances of *Bacteroidetes, Proteobacteria, Verrucomicrobia, Actinobcteria*, and *Fibrobacteres* increased, while that of *Firmicutes, Tenericutes*, and *Lentisphaerae* decreased substantially when reaching the perinatal period, respectively.

**Table 3:** The relative abundances of nine phyla (%, >1% in at least one sample) and *Firmicutes*/*Bacteroidetes* ratio during gestation.

	**Fat level**		**Inulin level**		**Gestation stage**				***P*****-value**					
	**Low**	**High**	**0**	**1.5%**	**d30**	**d60**	**d90**	**d110**	**F**	**I**	**S**	**F × I**	**F × S**	**I × S**
*Firmicutes*	51.80	53.31	51.96	53.15	54.76^b^	57.12^b^	58.08^b^	41.98^a^	0.40	0.51	<0.01	0.43	0.73	0.76
	[49.42; 54.30]	[50.94; 55.80]	[49.57; 54.46]	[50.78; 55.63]	[51.49; 58.24]	[53.71; 60.75]	[54.50; 61.90]	[39.39; 44.74]						
*Bacteroidetes*	29.50	26.97	28.40	28.01	26.27^a^	25.05^a^	27.28^a^	35.25^b^	0.06	0.76	<0.01	0.12	0.98	0.63
	[27.69; 31.43]	[25.37; 28.67]	[26.67; 30.25]	[26.35; 29.78]	[24.20; 28.53]	[23.07; 27.20]	[25.06; 29.71]	[32.38; 38.38]						
*Spirochaetes*	6.33	6.80	6.35	6.77	8.79^b^	8.09^b^	5.22^a^	4.99^a^	0.53	0.57	<0.01	0.69	0.44	0.07
	[5.41; 7.40]	[5.84; 7.91]	[5.43; 7.43]	[5.82; 7.89]	[7.25; 10.65]	[6.67; 9.81]	[4.28; 6.37]	[4.09; 6.08]						
*Proteobacteria*	2.42^b^	1.75^a^	2.06	2.06	1.41^a^	1.37^a^	2.51^b^	3.69^c^	0.04	0.99	<0.01	0.81	0.87	0.67
	[1.98; 2.95]	[1.44; 2.13]	[1.69; 2.51]	[1.69; 2.50]	[1.14; 1.74]	[1.11; 1.69]	[2.02; 3.13]	[2.97; 4.59]						
*Tenericutes*	2.92	3.57	3.66	2.85	3.86^b^	3.81^b^	3.12^b^	2.37^a^	0.16	0.09	<0.01	0.47	0.31	0.02
	[2.41; 3.54]	[2.95; 4.32]	[3.01; 4.43]	[2.36; 3.45]	[3.20; 4.66]	[3.15; 4.60]	[2.57; 3.78]	[1.95; 2.87]						
*Lentisphaerae*	0.49	0.41	0.35^a^	0.58^b^	1.09^b^	0.93^b^	1.07^b^	0.04^a^	0.37	0.02	<0.01	0.17	0.99	0.94
	[0.38; 0.64]	[0.32; 0.54]	[0.27; 0.46]	[0.44; 0.75]	[0.82; 1.44]	[0.70; 1.23]	[0.80; 1.42]	[0.03; 0.05]						
*Verrucomicrobia*	0.07	0.09	0.08	0.08	0.05^b^	0.03^b^	0.02^a^	1.68^c^	0.71	0.98	<0.01	0.37	0.06	0.47
	[0.06; 0.10]	[0.07; 0.11]	[0.06; 0.11]	[0.06; 0.11]	[0.03; 0.07]	[0.02; 0.05]	[0.01; 0.03]	[1.13; 2.49]						
*Fibrobacteres*	0.39	0.31	0.30	0.40	0.33^a^	0.34^ab^	0.24^a^	0.54^b^	0.41	0.28	0.02	0.43	0.48	0.62
	[0.27; 0.56]	[0.21; 0.44]	[0.21; 0.43]	[0.28; 0.57]	[0.22; 0.48]	[0.23; 0.50]	[0.16; 0.35]	[0.36; 0.81]						
*Actinobacteria*	0.21	0.27	0.26	0.22	0.20^a^	0.20^a^	0.18^a^	0.44^b^	0.08	0.24	<0.01	0.11	0.02	0.83
	[0.18; 0.25]	[0.22; 0.32]	[0.22; 0.31]	[0.19; 0.26]	[0.16; 0.25]	[0.16; 0.25]	[0.14; 0.23]	[0.34; 0.57]						
*Firmicutes/Bacteroidetes*	1.76	1.98	1.83	1.90	2.08^b^	2.28^b^	2.13^b^	1.19^a^	0.07	0.58	<0.01	0.13	0.94	0.87
	[1.60; 1.92]	[1.81; 2.16]	[1.67; 2.00]	[1.74; 2.07]	[1.84; 2.36]	[2.01; 2.58]	[1.87; 2.42]	[1.05; 1.35]						

*Data were log-transformed before statistical analysis, and hence confidence limits are given in brackets instead of SEM values. Sows were regarded as the experimental units, n = 5 for each treatment at each gestation stage except for LFD group with n = 4 on d90 and d110 of gestation due to an unexplained diarrhea on d88 of gestation which lasted for 2 days. When significant main effects or interative effects were observed, the means were compared using the least significant difference method with a P < 0.05 indicating significance. Therefore, within a row and within a main effect, mean values without a common letter are significantly different (P < 0.05). F, fat level; I, inulin level; S, gestation stage, F × I, interaction between fat and inulin level; F × S, interaction between fat and gestation stage; I × S, interaction between inulin and gestation stage. F × I × S interactions were tested but were not significant (P > 0.10)*.

### Changes of the relative abundance at genus level

The relative abundances at genus level (>1% in at least one sample) were present in Table 4. Fat addition did not show remarkable effects on many genera except that of *YRC22* was increased (*P* < 0.01) and that of *Prevotella* (*P* = 0.06) *and Terrisporobacter* (*P* = 0.07) tended to increase or decrease, respectively. Compared with fat addition, inulin addition showed much more prominent effects on the relative abundances of specific genera. Relative abundances of *Streptococcus* (*P* = 0.02), *Oscillospira* (*P* = 0.02), *Sphaerochaeta* (*P* = 0.02), and *Phascolarctobacterium* (*P* < 0.01) were increased while that of *YRC22* were reduced by inulin addition (*P* < 0.01). Interactive effects between fat and inulin levels were found for genera *Turicibacter* (*P* = 0.01), *Terrisporobacter* (*P* = 0.05), *YRC22* (*P* < 0.01), and *SMB53* (*P* < 0.01). Relative abundances of *Rikenellaceae_RC9_gut _group, Terrisporobacter*, and *Christensenellaceae_R-7_group* were not detected in fecal samples on d110 of gestation. By contrast, *Escherichi*a, *YRC22*, and *SMB53* were only detected in samples on d110 of gestation. The majority of genera were affected by gestation stage indicating that their relative abundances changed greatly over the pregnancy progress, in particular, when reaching the perinatal period.

**Table 4:** The relative abundances of nineteen genera (%, >1% in at least one sample) during gestation.

	**Fat level**		**Inulin level**		**Gestation stage**				***P*****-value**					
	**Low**	**High**	**0**	**1.5%**	**d30**	**d60**	**d90**	**d110**	**F**	**I**	**S**	**F × I**	**F × S**	**I × S**
*Treponema*	5.46	5.92	5.63	5.75	8.10^c^	7.64^c^	4.86^b^	3.48^a^	0.53	0.87	<0.01	0.96	0.49	0.13
	[4.57; 6.52]	[4.98; 7.04]	[4.71; 6.72]	[4.83; 6.83]	[6.58; 9.73]	[6.21; 9.41]	[3.92; 6.02]	[2.81; 4.32]						
*Clostridium*	6.79	6.64	6.52	6.91	9.40^b^	10.31^b^	10.07^b^	2.08^a^	0.80	0.52	<0.01	0.22	0.96	0.95
	[5.98; 7.70]	[5.87; 7.50]	[5.74; 7.40]	[6.11; 7.81]	[7.90; 11.18]	[8.67; 12.27]	[8.40; 12.04]	[1.74; 2.49]						
*Ruminococcus*	1.97	1.80	2.04	1.74	2.35^b^	2.85^b^	2.27^b^	0.83^a^	0.43	0.15	<0.01	0.10	0.43	0.82
	[1.69; 2.29]	[1.56; 2.09]	[1.75; 2.37]	[1.51; 2.02]	[1.91; 2.89]	[2.32; 3.52]	[1.83; 2.81]	[0.67; 1.02]						
*Prevotella*	1.36	2.03	1.62	1.70	1.48^b^	0.82^a^	1.57^b^	4.00^c^	0.06	0.80	<0.01	0.76	<0.01	0.08
	[1.03; 1.79]	[1.55; 2.65]	[1.23; 2.13]	[1.30; 2.23]	[1.10; 1.99]	[0.61; 1.09]	[1.16; 2.12]	[2.96; 5.41]						
*Streptococcus*	1.53	1.77	1.38^a^	1.97^b^	1.77^b^	2.00^b^	1.93^b^	1.07^a^	0.30	0.02	<0.01	0.60	0.69	0.43
	[1.25; 1.86]	[1.46; 2.15]	[1.13; 1.68]	[1.63; 2.38]	[1.40; 2.22]	[1.59; 2.52]	[1.52; 2.44]	[0.85; 1.36]						
*Oscillospira*	1.68	1.41	1.27^a^	1.87^b^	1.38^a^	1.40^a^	1.35^a^	2.16^b^	0.26	0.02	<0.01	0.59	0.02	0.60
	[1.37; 2.06]	[1.15; 1.73]	[1.03; 1.56]	[1.53; 2.29]	[1.12; 1.69]	[1.14; 1.72]	[1.10; 1.67]	[1.75; 2.66]						
*Sphaerochaeta*	0.38	0.40	0.31^a^	0.49^b^	0.49^b^	0.35^b^	0.28^a^	0.48^b^	0.76	0.02	0.01	0.11	0.98	0.50
	[0.30; 0.48]	[0.32; 0.51]	[0.24; 0.39]	[0.39; 0.62]	[0.37; 0.65]	[0.27; 0.46]	[0.21; 0.37]	[0.36; 0.64]						
*Turicibacter*	1.71	1.86	1.91	1.67	2.07^b^	2.34^b^	2.37^b^	0.88^a^	0.31	0.10	<0.01	0.01	0.09	0.62
	[1.54; 1.91]	[1.67; 2.06]	[1.71; 2.12]	[1.51; 1.85]	[1.79; 2.40]	[2.02; 2.70]	[2.04; 2.74]	[0.76; 1.03]						
*Phascolarctobacterium*	0.51	0.48	0.40^a^	0.61^b^	0.52	0.43	0.54	0.49	0.74	<0.01	0.52	0.12	0.59	0.10
	[0.42; 0.60]	[0.41; 0.58]	[0.33; 0.48]	[0.51; 0.73]	[0.42; 0.66]	[0.34; 0.55]	[0.43; 0.69]	[0.39; 0.62]						
*Lactobacillus*	0.56	0.51	0.60	0.48	0.59^b^	0.71^b^	0.59^b^	0.33^a^	0.60	0.24	<0.01	0.87	1.00	0.29
	[0.43; 0.73]	[0.40; 0.66]	[0.46; 0.78]	[0.37; 0.62]	[0.45; 0.79]	[0.54; 0.94]	[0.44; 0.78]	[0.25; 0.44]						
*Rikenellaceae_RC9_gut_group*	3.94	3.60	3.86	3.67	4.76^c^	3.82^b^	2.94^a^	ND	0.12	0.38	<0.01	0.30	0.22	0.15
	[3.64; 4.27]	[3.33; 3.89]	[3.57; 4.18]	[3.39; 3.97]	[4.32; 5.24]	[3.47; 4.20]	[2.66; 3.24]							
*Terrisporobacter*	3.24	2.77	2.76^a^	3.25^b^	2.68	3.20	3.13	ND	0.07	0.07	0.14	0.05	0.95	0.82
	[2.89; 3.63]	[2.48; 3.10]	[2.47; 3.10]	[2.90; 3.63]	[2.34; 3.06]	[2.80; 3.65]	[2.73; 3.59]							
*Fibrobacter*	0.39	0.31	0.30	0.40	0.33^a^	0.34^a^	0.24^a^	0.54^b^	0.41	0.28	0.02	0.44	0.48	0.63
	[0.27; 0.56]	[0.21; 0.44]	[0.21; 0.43]	[0.28; 0.57]	[0.22; 0.48]	[0.23; 0.50]	[0.16; 0.35]	[0.36; 0.81]						
*Bacteroides*	0.88	0.80	0.82	0.86	0.82^b^	0.92^b^	0.64^ab^	1.02^c^	0.41	0.64	<0.01	0.65	0.88	0.29
	[0.76; 1.01]	[0.70; 0.93]	[0.71; 0.95]	[0.75; 0.99]	[0.69; 0.97]	[0.78; 1.10]	[0.54; 0.77]	[0.85; 1.21]						
*Christensenellaceae_R-7_group*	1.62	1.65	1.65	1.62	1.55^a^	1.86^b^	1.52^a^	ND	0.75	0.74	<0.01	0.53	0.91	0.82
	[1.51; 1.75]	[1.54; 1.77]	[1.53; 1.78]	[1.51; 1.74]	[1.44; 1.68]	[1.72; 2.01]	[1.40; 1.64]							
*Bifidobacterium*	0.04	0.03	0.04	0.04	0.04	0.04	0.03	0.04	0.07	0.79	0.09	0.35	0.43	0.05
	[0.03; 0.05]	[0.03; 0.04]	[0.03; 0.04]	[0.03; 0.05]	[0.03; 0.05]	[0.03; 0.05]	[0.02; 0.04]	[0.03; 0.05]						
*Escherichia*	0.46	0.35	0.29	0.55	–	–	–	0.40	0.63	0.26	–	0.62	–	–
	[0.21; 0.99]	[0.17; 0.72]	[0.14; 0.63]	[0.27; 1.13]				[0.24; 0.68]						
*YRC22*	0.95^a^	1.75^b^	1.64^b^	1.01^a^	–	–	–	1.29	<0.01	<0.01	–	<0.01	–	–
	[0.76; 1.17]	[1.43; 2.15]	[1.32; 2.03]	[0.83; 1.24]				[1.11; 1.49]						
*SMB53*	1.87	1.79	1.71	1.96	–	–	–	1.83	0.76	0.39	–	<0.01	–	–
	[1.51; 2.32]	[1.46; 2.19]	[1.39; 2.12]	[1.60; 2.39]				[1.58; 2.12]						

*Data were log-transformed before statistical analysis, and hence confidence limits are given in brackets instead of SEM values. Sows were regarded as the experimental units, n = 5 for each treatment at each gestation stage except for LFD group with n = 4 on d90 and d110 of gestation due to an unexplained diarrhea on d88 of gestation which lasted for 2 days. When significant main effects or interative effects were observed, the means were compared using the least significant difference method with a P < 0.05 indicating significance. Therefore, within a row and within a main effect, mean values without a common letter are significantly different (P < 0.05). F, fat level; I, inulin level; S, gestation stage, F × I, interaction between fat and inulin level; F × S, interaction between fat and gestation stage; I × S, interaction between inulin and gestation stage; ND, not detected. F × I × S interactions were tested but were not significant (P > 0.10)*.

### Changes of the relative abundance of representative VFA-producing related genera

**Figure 5:**
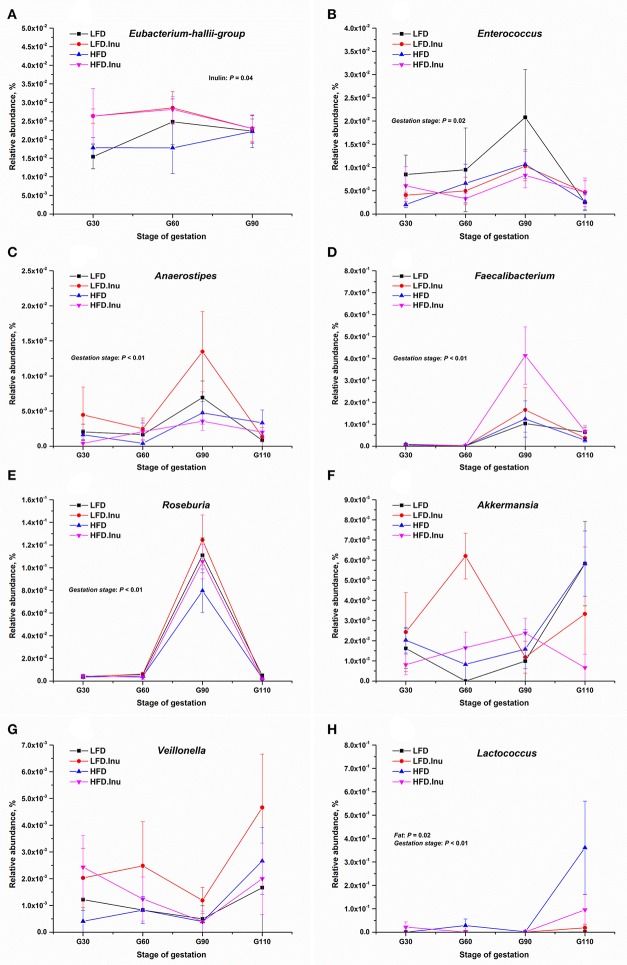
The relative abundances of representative VFA-producing related genera (%) during gestation. **(A)** *Eubacterium-hallii-group*: *P* = 0.04 for inulin effect. **(B)** *Enterococcus*: *P* = 0.02 for gestation stage. **(C)** *Anaerostipes*: *P* < 0.01 for gestation stage. **(D)** *Faecalibacterium*: *P* < 0.01 for gestation stage. **(E)** *Roseburia*: *P* < 0.01 for gestation stage. **(F)** *Akkermansia*. **(G)** *Veillonella*. **(H)** *Lactococcus*: *P* = 0.02 for fat effect and *P* < 0.01 for gestation stage. Data were log-transformed following the addition of a small offset (0.00001) to counteract the presence of zero values before statistical analysis. Sows were regarded as the experimental units, *n* = 5 for each treatment at each gestation stage except for LFD group with *n* = 4 on d90 and d110 of gestation due to an unexplained diarrhea on d88 of gestation which lasted for 2 days. When significant main effects or interative effects were observed, the means were compared using the least significant difference method with a *P* < 0.05 indicating significance. LFD, low fat diet; LFD.Inu, low fat diet with inulin addition; HFD, high fat diet; HFD.Inu, high fat diet with inulin addition. Only significant *p*-values were presented in the figure.

As shown in Figure 5, fat addition markedly increased the abundance of *Lactococcus* (*P* = 0.02). Despite numerical increases were found for most VFA-producing genera by inulin addition, only genus *Eubacterium-hallii-group* significantly increased (*P* = 0.04). Remarkable increase of *Bacteroides* (Table 4; *P* < 0.01) whose main fermentative product is propionate, and notable decreases of *Roseburia* and *Anaerostipes* (*P* < 0.01) whose main fermentative products are butyrate were detected from d90 of gestation to the perinatal period. Abundance changes of *Enterococcus* and *Lactococcus* whose main fermentative products are lactate showed the opposite way with *Enterococcus* decreasing and *Lactococcus* increasing from d90 of gestation to the perinatal period.

## Discussion

Rodents have been used frequently as animal models for humans, however, the discrepancies in physiology and metabolism compared with humans are worth considering. In contrast, the similarities between humans and pigs in terms of anatomic, cardiovascular, gastrointestinal systems place the pig in a superior position over other non-primate models in many studies (Swindle et al., 2012). Above all, the pig is a human-sized omnivorous animal with comparable requirements for nutrients, therefore shows similarities to the human intestinal microbial ecosyste (Heinritz et al., 2013). This has driven the pig to be the alternative animal model for research into dietary modulation of microbiota–health interactions.

It is well-known that excessive intake of dietary fat is associated with being overweight or obese while dietary fiber has been extensively demonstrated to be an effective dietary intervention mean to reduce body weight gain and fat accumulation (Galisteo et al., 2005; Han et al., 2015, 2017; Palou et al., 2015). Although there is a scarcity of evidence on the effects of dietary fat or fiber in pregnant individuals, our findings that inulin attenuated the high fat diet-induced body weight gain and fat mass accumulation were in good agreement with previous studies conducted on non-pregnant individuals (Yan et al., 2013; Alligier et al., 2014; Cluny et al., 2015).

To evaluate the potential effects of lower gestational body weight and backfat gain induced by inulin supplementation, concentrations of circulating inflammatory indices and adipocytokines in both circulation and backfat tissues were investigated. Generally, obesity and related metabolic disorders are characterized by chronic or low-grade inflammation (Hotamisligil, 2006; Saltiel and Olefsky, 2017). Weight loss or fat mass reduction has been proven to result in reduction of inflammatory biomarkers (Richard et al., 2013; Barazzoni et al., 2014). Unsurprisingly, significantly decreased IL-6 and numerically increased IL-10 with inulin addition in the present study indicated an alleviative effect of inulin on maternal inflammatory status at perinatal period. The anti-inflammatory effect of inulin was also confirmed by Yasuda et al. (2009) and Lecerf et al. (2012) previously.

Leptin, adiponectin, and chemerin are adipocyte-secreted hormones with different physiological effects. Leptin regulates a wide variety of physiological processes, including feeding behavior, metabolic rate, sympathetic nerve activity, reproduction and immune response (Xiong et al., 2004; Brüll et al., 2017). Acute oral administration of specific SCFA *in vivo*, or incubating adipocyte cell line or adipose tissue with SCFAs *in vitro* have resulted in increased circulating leptin levels (Xiong et al., 2004) or leptin expression (Xiong et al., 2004; Al-Lahham et al., 2010). The mechanism under which SCFAs stimulate leptin production was due to the activation of G protein-coupled receptors (GPCRs) expression in adipose tissues by SCFAs which acts as the specific agonists of GPCRs (Xiong et al., 2004; Zaibi et al., 2010). In sharp contrast to those findings, there was consistent data showing that chronic HFD resulted in increased, while chronic dietary fiber addition caused decreased level of serum leptin in both non-pregnant (Artiss et al., 2006; Islam et al., 2011) and pregnant individuals (Vähämiko et al., 2013). The discrepancies among studies could be explained by the *in vivo* or *in vitro*, chronic or acute experimental backgrounds. Circulating leptin levels are directly associated with adipose tissue mass (Campfield et al., 1996; Ghantous et al., 2015). It is noteworthy that the decreased serum leptin level in chronic dietary manipulation *in vivo* studies were generally accompanied by distinctly less body weight gain when compared with high fat–fed group. As a consequence, despite the well-known leptin-stimulating effect in adipose tissue, circulating leptin level could still exert obvious reduction due to the less body weight gain and body fat accumulation with inulin addition combined with a high-fat diet. It should be mentioned that highly elevated leptin levels during pregnancy are associated with maternal leptin resistance, insulin resistance, metabolic disturbance, and increased risk of gestational diabetes and hypertension (Butte, 2000; Heerwagen et al., 2010; Vähämiko et al., 2010). Adiponection has anti-inflammatory and insulin-sensitizing effects. Plasma adiponectin concentration is negatively associated with adiposity (Nakamura et al., 2014). Although a growing body of evidence has indicated the positive association between blood adiponectin level and dietary fiber intake (Yannakoulia et al., 2008a,b; AlEssa et al., 2016), we only found a tendency of increase in adiponectin level in backfat tissue, but not in the circulating concentration. Chemerin, a recently identified adipokine that regulates adipocyte differentiation, has been positively linked to adiposity, insulin resistance, metabolic syndrome risk factors, and inflammatory markers (Lehrke et al., 2009; Ernst and Sinal, 2010; Sell et al., 2010; Weigert et al., 2010). Previous studies have indicated circulating levels of chemerin correlated with BMI, and weight loss was able to decrease the circulating chemerin level and adipose tissue *chemerin* expression in humans (Sell et al., 2010; Chakaroun et al., 2012). In line with these findings, circulating and backfat tissue chemerin levels in our study were significantly reduced by inulin addition, which may contribute to improved insulin sensitivity and subclinical inflammation beyond significant weight loss as indicated by Chakaroun et al. (2012).

As we know, fetal growth and development is primarily dependent upon the nutritional, hormonal and metabolic environment provided by the mother (Tzanetakou et al., 2011). Previous studies have indicated that maternal high fat feeding could cause obesity in dams and modulate the intrauterine environment to predispose offspring to obesity and related metabolic disorders (Shankar et al., 2008; White et al., 2009). Strikingly, along with the alleviation of maternal body weight gain and fat accumulation as well as changes in inflammatory markers and metabolic hormones, inulin addition resulted in lower average BMI and a more even BMI distribution of the neonatal offspring, and a decreased probability of relatively “obese” newborns.

Although multiple mechanisms have been suggested to involve in such positive regulation of DF in maternal body mass and metabolic status, the central role of gut microbiota in development of obesity and its associated inflammation and metabolic disturbance has come to the forefront (Bäckhed et al., 2004; Turnbaugh et al., 2006, 2009; Shen et al., 2013). An in-depth insight into the shift of microbiota composition with inulin supplementation during gestation would provide us a novel understanding about the beneficial effect of inulin on regulation of body weight, inflammatory status and adipose tissue derived hormones which have great impact on host metabolism.

Various factors including host genetics, diets, immunological status, and antibiotic use could shape the gut microbiota (Koren et al., 2012; Scott et al., 2013). Due to the preference for different energy sources, the gut microbiota can undergo dynamic population shifts with varied dietary manipulation even during pregnancy (Gohir et al., 2015; Kong et al., 2016).

Low microbial diversity is often associated with diseases such as inflammatory bowel disease (Manichanh et al., 2006) and obesity (Turnbaugh et al., 2009). In the present study, the significant increase in Simpson index with inulin addition, particularly when given with a high-fat diet was in good agreement with Li et al. (2016) who found Shannon's richness index was significantly decreased in high-fat feeding mice and restored under bamboo fiber addition. Therefore, the improved microbial diversity would partly explain the reduction in gestational weight and backfat gain as well as the improved inflammation response at perinatl period.

The abundant phyla in sow feces were in good agreement with previous studies on pigs (Kong et al., 2016; Yan et al., 2016) with *Firmicutes* and *Bacteroidetes* accounting for 75% or more of the microbial composition. Previous studies had demonstrated HFD could increase *Firmicutes* and decrease *Bacteroidetes* abundance (Turnbaugh et al., 2008). Increased ratio of *Firmicutes* to *Bacteroidetes* was indicated to be associated with obesity phenotype (Ley et al., 2005). In line with the tendency of increase in *Firmicutes*/*Bacteroidetes*, we did found notable increases in maternal weight and backfat gain with fat addition. Phylum *Actinobacteria* has been shown to be substantially more abundant in inflammatory bowel disease patients (Frank et al., 2007) and in colon and terminal ileum of obese Ossabaw minipigs (Pedersen et al., 2013) than their control counterparts. The tendency of increase of phylum *Actinobacteria* with high fat supplementation coincided with the excessive gestational weight gain induced by fat addition to some extent, also suggested the low-grade inflammatory state of sows under high fat treatment. Phylum *Tenericutes*, similarly, has been found to have significant higher abundance in obese Göttingen pig cecum (Pedersen et al., 2013) and diet-induced obese mice (Turnbaugh et al., 2008) compared to the controls. On the contrary, phylum *Lentisphaerae* was demonstrated to be higher in the healthy group than in humans with non-alcoholic fatty liver disease (NAFLD) (Jiang et al., 2015). The tendency of decrease in *Tenericutes* with inulin addition were partially in agreement with results found by Everard et al. (2014) who found probiotic yeast addition decreased 57% of the amount of *Tenericutes*. Moreover, significantly higher abundance of *Lentisphaerae* due to inulin addition in the present study would also be another possible microbial mechanism underlying the regulatory effects of inulin on host body weight and fat storage.

*Oscillospira* has been observed to relate to, even contribute to leanness or lower BMI and also negatively be associated with inflammatory diseases as reviewed by Konikoff and Gophna (2016). The potential underlying mechanism, is due to the fact that genus *Oscillospira* was able to degrade mammalian-derived glycans (such as fucose, sialic acids, and glucuronic acid) either from the host or from diet, leading to extra metabolic energy consumption by host to regenerate degraded glycoproteins that compose, for example, intestinal mucins (Kohl et al., 2014). Significant increased abundance of *Oscillospira* with inulin addition could explain the body weight- and fat storage-reducing effects of inulin at genus level. Genus *Streptococcus* was found to involve in the fermentation of sugars, yielding lactic acid as their predominant fermentation end product (van den Bogert et al., 2013). Species belonging to *Phascolarctobacterium* (for e.g., *Phascolarctobacterium succinatutens*) were demonstrated to be able to further metabolize succinate, a fermentation product of dietary fiber, to form propionate (Engels et al., 2016). The increases of these two genera well confirmed the nature of inulin as a dietary fiber to be utilized by specific bacteria. Compared with inulin, fat addition showed less dramatic effects on the modification of genus taxonomies. As the most well-known probiotics, *Lactobacillus* and *Bifidobacterium* have received great concern in previous studies. It should be mentioned that there were no significant increases for both of them with inulin addition in our study. This missing effect was in line with results reported by Paßlack et al. (2015) with 3% inulin addition in sows during gestation and lactation and results obtained by Mair et al. (2010) with 0.4% inulin addition in newly weaned piglets.

With regard to different gestation stages, our present results were inconsistent with Koren et al. (2012) and Kong et al. (2016) who found decreased OTUs in pregnant women or pigs, as well as Collado et al. (2008) who found increased total fecal cell counts from the first to third trimester in women. The discrepancy could be contributed by the genetic background of the experimental subjects and environmental deviation. Slight but not significant decline in Simpson index over the gestation aligned with the results obtained by Kong et al. (2016) and DiGiulio et al. (2015). However, significant increase of Chao 1 index on d110 of gestation, which is some controversial to previous results which found decreased Chao 1 index in sows (Kong et al., 2016) and similar Chao 1 index in women (DiGiulio et al., 2015) from early to late of gestation, was mostly caused by fat and inulin addition in the present study. This finding underscores the efficacy of different dietary treatments on modifying the gut microbiota.

At either phylum or genus level, results obtained on d110 of gestation showed striking differences from other gestation stages. Decreased relative abundance of *Firmicutes*, increased relative abundance of *Bacteroidetes*, as well as decreased *Firmicutes/Bacteroidetes* ratio on d110 of gestation was in line with results reported in the proximal colon content samples by Kong et al. (2016) who sampled on d45, d75, and d110 of gestation, however, Koren et al. (2012) and DiGiulio et al. (2015) found no dramatic change over the course of gestation in women. According to Jumpertz et al. (2011), increase in *Firmicutes* and a corresponding decrease in *Bacteroidetes* were associated with an increased energy harvest. Our results indicated some degree of increased energy expenditure on d110 of gestation. In fact, viewed from genus level, the decreased relative abundances of *Clostridum, Ruminococcus, Turicibacter, Lactobacillus, Terrisporobacter*, and *Christensenellaceae_R-7_group*, along with the increased abundances of *Prevotella, Bacteroides*, and *YRC22* on d110 of gestation could well explain the decline in *Firmicutes* and increment in *Bacteroidetes*, respectively. Most species of *Ruminococcus* fall under *Clostridium cluster IV* and *Clostridium cluster XIVa*, which have been associated with obesity, weight gain and lipid storage (Turnbaugh et al., 2006; Nadal et al., 2009). The abrupt decline of *Clostridum*, more than 50% decline of *Ruminococcus* as well as decreases of other genera on d110 of gestation indicated that the sow were in catabolic metabolic state during the perinatal period. It is well demonstrated previously that body fat stores and protein reserves mobilized evidently at perinatal period due to reallocation of nutrients from the conceptus to the mammary tissue during which massive mammary growth occurs, and colostrum production is being initiated (Theil et al., 2014). Correspondingly, heat production also increases during this time (Theil, 2015). These physiological changes contribute to the shift of maternal metabolism from an anabolic to a catabolic state. The findings that specific microbial communities changed dramatically at perinatal period may provide the first evidence to reveal the microbial mechanism under which maternal catabolic state appeared around parturition.

Our results were in partially accordance with previous studies proving that gut microbiota changed greatly from first to third trimester of gestation with increased abundances of *Proteobacteria* and *Actinobacteria* (Koren et al., 2012; Gomez-Arango et al., 2016). Furthermore, the genus *Escherichia* belonging to *Proteobacteria* was only detected on d110 of gestation in the present study. *Proteobacteria* is a phylum known to encompass multiple pathogens and have pro-inflammatory properties (Mukhopadhya et al., 2012); in particular, some pathogenic *Escherichia coli* falling under the genus *Escherichia* are known to induce a series of inflammation responses. Moreover, a number of species from phylum *Actinobacteria* were associated with obesity (Turnbaugh et al., 2009). It is common recognized that the latter stage of gestation is characterized by a diabetogenic state in the mother to support the continuous supply of nutrients to the fetus, and an elevated levels of circulating proinflammatory cytokines (Mor and Cardenas, 2010). Increases of these two phyla provide us the insight into the microbial mechanism under which the mother performs some degree of inflammation during late gestation. Meanwhile, it confirms the previous conclusion that the gut microbial composition in the first trimester of pregnancy is similar to that of healthy, non-pregnant women (Nuriel-Ohayon et al., 2016), whereas that in the third trimester resembled an insulin resistance and greater inflammatory response–associated dysbiosis (Koren et al., 2012).

Fermentation of dietary fiber in the hindgut results in multiple groups of metabolites of which SCFAs are the major products. Acetic acid (C2), propionic acid (C3), and butyric acid (C4) are the most abundant, representing 90–95% of the SCFA present in the colon (Ríos-Covián et al., 2016). During the intestinal absorption process, butyrate, mainly acting as the energy source, is metabolized by the colonocytes whilst the rest will be transported to the liver and be used in different biosynthetic routes. Propionate will mainly act as a precursor for gluconeogenesis (Roy et al., 2006), acetate and butyrate will be mostly introduced into the lipid biosynthesis (den Besten et al., 2013b; Ríos-Covián et al., 2016). In addition to being as substrates, SCFA can act as signaling molecules to be sensed by specific G protein –coupled receptors (GPRs) and involve in the regulation of lipid and glucose metabolism (den Besten et al., 2013a). All these contribute to the central role of SCFA in the diet–gut microbiome-host metabolism axis. Due to the fact that acetate is produced by most enteric bacteria as a product of carbohydrate fermentation, it is the most abundant fecal VFA and makes up more than 50% of the total detected in feces in our study which is in good agreement with Louis et al. (2007). The linear decreases of individual and total VFA concentrations from d30 to d90 of gestation were partially in line with those reported by Kong et al. (2016) who found acetate and total SCFAs have a tendency to decrease as the pregnancy progressed, however, contradictory results with remarkable increases on d110 of gestation were also found in our study. This might imply a greater amount of energy loss in feces at perinatal period as suggested by Koren et al. (2012) who did find significant increase in stool energy content in T3 compared with T1 trimester. The possible explanation for the reduced metabolite absorption would be the potentially decreased GIT contractility. Studies had suggested the progressively rises in pregnancy hormones such as progesterone and abdominal compression from the enlarging gravid uterus during gestation would lead to decreased GIT contractility (Body and Christie, 2016). In comparison to a growing body of evidence indicating that high fiber–LFD are characterized by the presence of higher amounts of fecal SCFA than diets with lower fiber content (De Filippo et al., 2010; Cuervo et al., 2013; Heinritz et al., 2016), our results align with those obtained by Paßlack et al. (2015) who did not detect any significant effects of inulin inclusion on total SCFA and its constituents in feces of sows during gestation and lactation. Furthermore, Nyman (2002) reviewed that inulin-type fructans generally does not lead to a significant increase of fecal SCFA or to a change in molar proportions of acetate, propionate, and butyrate, which can be explained by their very efficient colonic absorption in humans with <5% being excreted in feces (Raninen et al., 2011). It should be mentioned here that the lacked effects of inulin addition on the fecal VFA concentrations would question whether stool SCFA output is a suitable proxy for luminal SCFA production as introduced by Vogt and Wolever (2003). Previously, fecal SCFA concentrations had been suggested to be more suitable to reflect the overall net production and absorption of SCFAs in the GIT, rather than the production or the absorption of SCFAs *per se* (Vogt and Wolever, 2003; Montoya et al., 2016). To evaluate the potential production of VFAs, abundances of VFA producing-related genera were studied. Despite only the butyrate-producing genus *Eubacterium-hallii-group* was significantly increased by inulin addition, an overwhelming majority of presented genera were numerically increased or showed tendency to increase. With respect to the effect of gestation stage, propionate-producing genera and butyrate-producing genera showed the opposite way with the former increasing and the latter decreasing from d90 of gestation to perinatal period. The mechanism underlying the discrepancy between the abundance changes of propionate- and butyrate-producing genera is not clear yet, but may be related to the fate of these two VFAs in the host metabolism at perinatal period.

In conclusion, the present study suggested that improved microbial dysbiosis triggered by soluble dietary fiber inulin addition during gestation would be the potential mechanism underlying the positive effect of inulin on gestational weight gain regulation, maternal anti-inflammation and adipose tissue derived hormone production, as well as the lower neonatal average BMI and the lower risk of relatively “obese” newborns. Dramatical changes of energy metabolism- and inflammation-related microbial communities and their metabolites VFAs indicated a catabolic and inflammatory state under which the mother was undergoing at perinatal period. Given that surprisingly little understanding about the maternal microbial changes and corresponding metabolic improvement induced by soluble DF supplementation during gestation exists at present, special focus should be given when further studies are to be conducted. Application of soluble DF inulin in gestational diet would be a fruitful area for prevention of diet induced excess gestational weight gain and related inflammation status and metabolic disturbances with remodeled microbial ecosystem.

## Author contributions

LC, JL, YLin, SX, BF, ZF, and DW contributed to the experimental design. PZhou, YZ, YLi, and TG performed the animal experiments. PZhou, PZhang, and CJ executed the lab analysis. PZhou and YZ performed the statistical analysis and wrote the manuscript. JW and DW revised the manuscript. All authors read and approved the final manuscript.

### Conflict of interest statement

The authors declare that the research was conducted in the absence of any commercial or financial relationships that could be construed as a potential conflict of interest.
