# Supplementary material for: Microbial Mechanistic Insight into the Role of Inulin in Improving Maternal Health in a Pregnant Sow Model
Source: Front Microbiol. 2017 Nov 17;8:2242. doi: 10.3389/fmicb.2017.02242 (PMC5698696; doi:10.3389/fmicb.2017.02242)
Supplement: Supplementary file 1 [file DataSheet1.docx]

Supplementary Material

**Microbial Mechanistic Insight into the Role of Inulin in Improving Maternal health in a Pregnant Sow Model**

**Pan Zhou, Yang Zhao, Pan Zhang, Yan Li, Taotao Gui, Jun Wang, Chao Jin, Lianqiang Che, Jian Li, Yan Lin, Shengyu Xu, Bin Feng, Zhengfeng Fang, De Wu***

***Correspondence:** De Wu**:** sow_nutrition@sina.com

**Table S1.** Ingredient and nutrient composition of gestational diets (as-fed basis)

| Item | LFD | LFD.Inu |  | HFD | HFD.Inu |
| --- | --- | --- | --- | --- | --- |
| Ingredient, % |  |  |  |  |  |
| Corn | 55.83 | 55.83 |  | 55.83 | 55.83 |
| Soybean meal, | 15.12 | 15.12 |  | 15.12 | 15.12 |
| Wheat bran | 18.50 | 18.50 |  | 18.50 | 18.50 |
| Soybean oil^1^ | - | - |  | 5.00 | 5.00 |
| Maize starch | 6.50 | 5.00 |  | 1.50 | - |
| Inulin | - | 1.50 |  | - | 1.50 |
| L-Lysine (98.5%) | 0.04 | 0.04 |  | 0.04 | 0.04 |
| DL-Methionine (99%) | 0.05 | 0.05 |  | 0.05 | 0.05 |
| L-Threonine (98.5%) | 0.06 | 0.06 |  | 0.06 | 0.06 |
| Calcium carbonate | 1.11 | 1.11 |  | 1.11 | 1.11 |
| Monocalcium phosphate | 1.69 | 1.69 |  | 1.69 | 1.69 |
| Choline | 0.15 | 0.15 |  | 0.15 | 0.15 |
| Salt | 0.40 | 0.40 |  | 0.40 | 0.40 |
| Vitamin premix^2^ | 0.05 | 0.05 |  | 0.05 | 0.05 |
| Trace mineral premix^3^ | 0.50 | 0.50 |  | 0.50 | 0.50 |
|  |  |  |  |  |  |
| Nutrient composition |  |  |  |  |  |
| Digestible energy^4^, Mcal/kg | 3.07 | 3.01 |  | 3.31 | 3.25 |
| Crude protein, % | 13.80 | 13.79 |  | 13.78 | 13.78 |
| Crude fat, % | 2.99 | 2.99 |  | 7.88 | 7.88 |
| Crude fiber,% | 3.04 | 4.54 |  | 3.04 | 4.54 |
| Lysine, % | 0.66 | 0.66 |  | 0.66 | 0.66 |
| Methionine + Cysteine, % | 0.41 | 0.41 |  | 0.41 | 0.41 |
| Threonine, % | 0.47 | 0.47 |  | 0.47 | 0.47 |
| Tryptopan, % | 0.13 | 0.13 |  | 0.13 | 0.13 |
| Ca, % | 0.90 | 0.90 |  | 0.90 | 0.90 |
| Available P, % | 0.45 | 0.45 |  | 0.45 | 0.45 |

^1^Main fatty acids composition (g/100g oil): palmitic acid (C16:0), 9.73; stearic acid (C18:0), 4.02; oleic acid (C18:1), 21.72; linoleic acid (C18:2), 53.71; linolenic acid (C18:3), 6.79.

^2^Provided per kg of diet: 25,000 IU vitamin A; 5,000 IU vitamin D3; 12.5 IU vitamin E; 2.5 mg vitamin K; 1 mg vitamin B1; 8 mg vitamin B2;3 mg vitamin B6; 0.015 mg vitamin B12; 17.5 mg niacin; 12.5 mg pantothenic acid; 0.25 mg folacin.

^3^Provided per kg of diet: 165 mg Fe; 16 mg Cu; 165 mg Zn; 30 mg Mn; 0.3 mg Se; 0.3 mg I.

^4^Inulin could generate energy by fermentation, but our digestible energy did not account for this part of energy.

**Table S2.** Average raw reads, effective tags and OTUs of fecal microbial community during gestation

|  | Average raw reads | Average effective tags | Average OTUs |
| --- | --- | --- | --- |
| D 30 of gestation |  |  |  |
| LFD | 76641 | 73924 | 1381 |
| LFD.Inu | 73350 | 71077 | 1206 |
| HFD | 78678 | 76151 | 1208 |
| HFD.Inu | 81786 | 79292 | 1201 |
| D 60 of gestation |  |  |  |
| LFD | 77957 | 75682 | 1232 |
| LFD.Inu | 76480 | 74245 | 1241 |
| HFD | 80085 | 77444 | 1232 |
| HFD.Inu | 74778 | 72155 | 1237 |
| D 90 of gestation |  |  |  |
| LFD | 75712 | 73306 | 1241 |
| LFD.Inu | 78065 | 75704 | 1216 |
| HFD | 85559 | 82795 | 1259 |
| HFD.Inu | 80205 | 77472 | 1284 |
| D 110 of gestation |  |  |  |
| LFD | 53720 | 50657 | 1225 |
| LFD.Inu | 63022 | 60597 | 1451 |
| HFD | 54842 | 52041 | 1778 |
| HFD.Inu | 61283 | 58550 | 1536 |

LFD, low fat diet; LFD.Inu, low fat diet with inulin addition; HFD, high fat diet; HFD.Inu, high fat diet with inulin addition.

**A**

**

**

**B**

**

**

**C**

**

**

**D**

**

**

**E**

**

**

**Figure S1.** (**A**) Fecal total VFA and (**B**-**D**) individual VFA concentration and (**E**) PH value over the course of gestation. Values without a common letter are significantly different (*P* < 0.05). **P* < 0.05, ***P* < 0.01. LFD, low fat diet; LFD.Inu, low fat diet with inulin addition; HFD, high fat diet; HFD.Inu, high fat diet with inulin addition.

**A B**


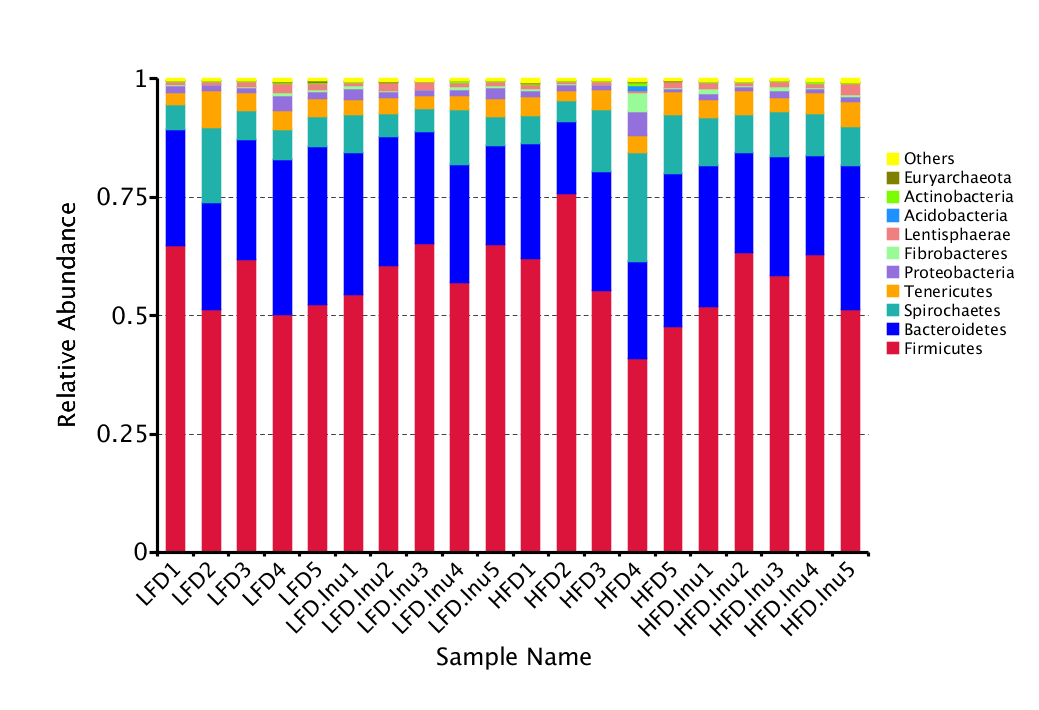


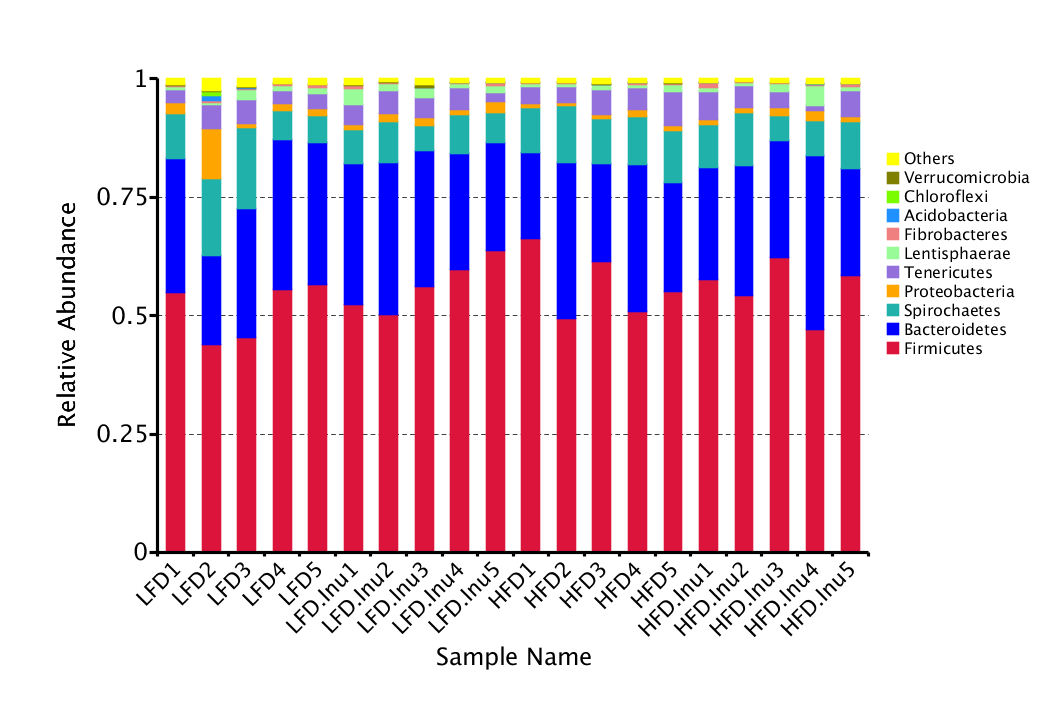


**C D**


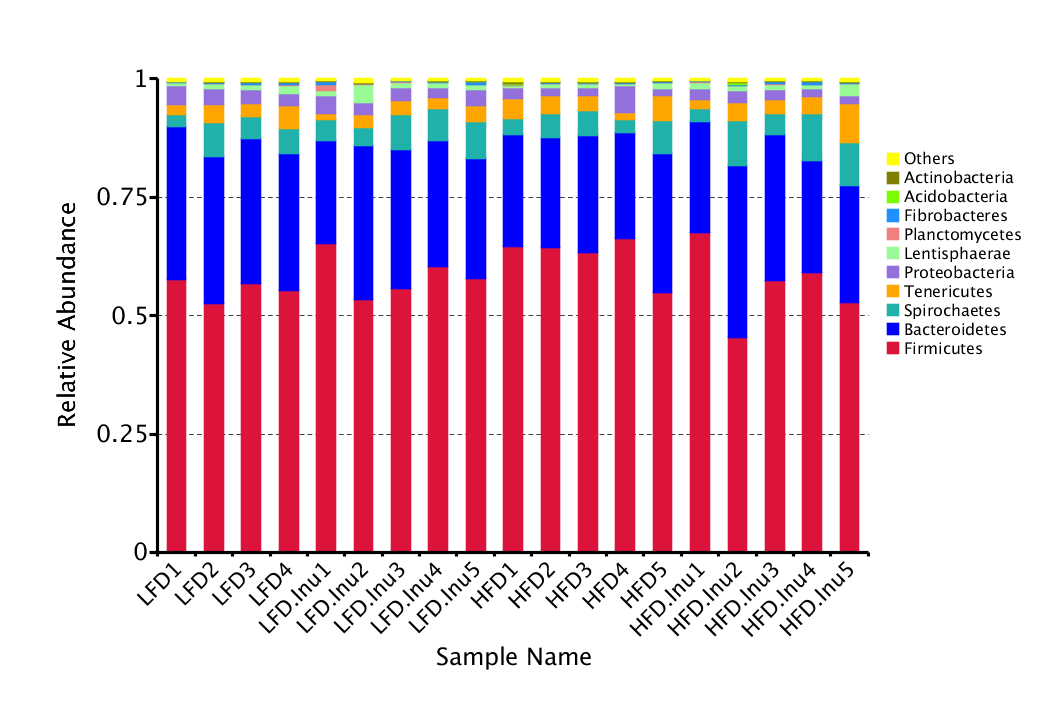

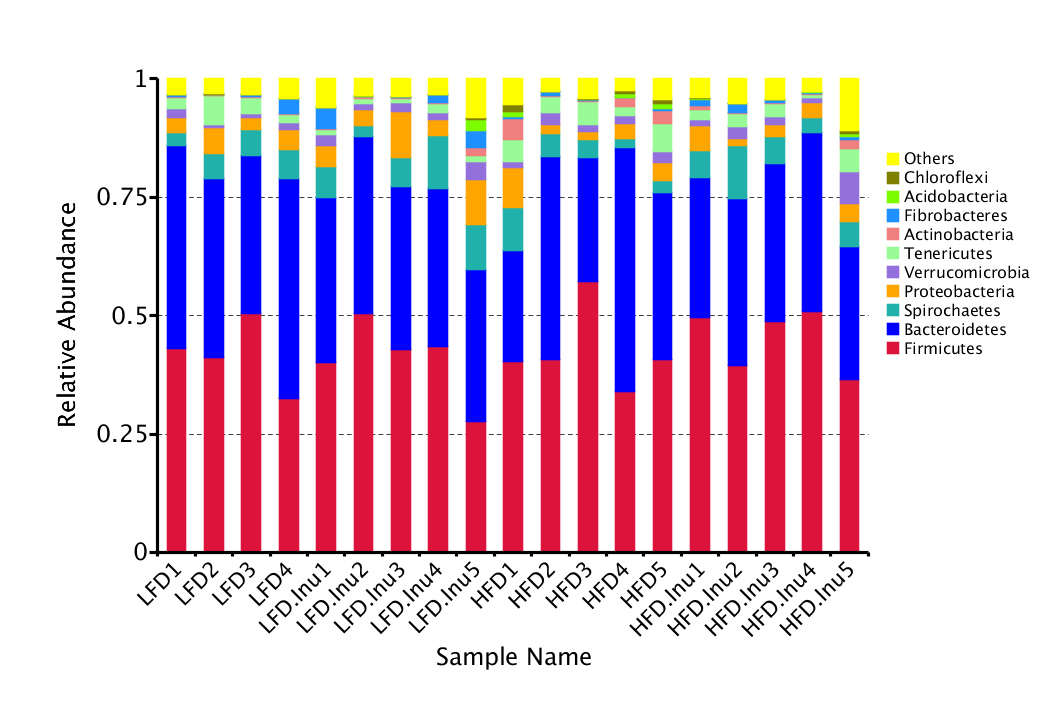


**Figure S2.** The relative abundance at phylum level at each stage of gestation. (**A**) d30 of gestation. (**B**) d60 of gestation. (**C**) d90 of gestation. (**D**) d110 of gestation. LFD, low fat diet; LFD.Inu, low fat diet with inulin addition; HFD, high fat diet; HFD.Inu, high fat diet with inulin addition.
